# Optimizing learning in undergraduate psychology students: the impact of advance quizzing, review, and classroom attendance

**DOI:** 10.1186/s41235-017-0075-2

**Published:** 2017-09-20

**Authors:** Beat Meier

**Affiliations:** Institute of Psychology and Center for Cognition, Learning, and Memory, University of Bern, Fabrikstr. 8, 3012 Bern, Switzerland

**Keywords:** Memory, Testing effects, Pretesting effects, Podcasts

## Abstract

This study investigates whether introducing simple cognitive interventions that are known to enhance learning in laboratory studies can be transferred to classroom settings. In an introductory psychology class, students were provided with a brief advance quiz on the topic covered in each particular lecture. In case they did not attend class, they still had the opportunity to fill it out at home. The interventions were offered on a voluntary basis and the students were given the opportunity to obtain an extra point towards the final exam by reviewing the key points of each lecture on a regular basis. The results indicate that both advance quizzing and review enhanced the grades on the final examination. Although it is likely that individual differences also contribute to learning outcomes, these effects were specific for the particular course and independent from class attendance. The results indicate that transfer of simple cognitive interventions from laboratory to classroom settings is feasible. They also indicate that these interventions can be effectively implemented into the lectures to boost student learning.

**Electronic supplementary material:**

The online version of this article (doi:10.1186/s41235-017-0075-2) contains supplementary material, which is available to authorized users.

## Significance

Undergraduate university students have only a fragmentary knowledge about effective learning strategies. In contrast, cognitive psychologists have a very fine-grained understanding about strategies and activities that benefit learning in laboratory studies. One of the most important insights is the effect of retrieval as an effective learning strategy. Retrieval benefits learning even for materials to be studied but not yet studied (i.e., advance quizzing). Retrieval induced as a review of key points between lectures also benefits learning. Transferring these strategies and activities into the classroom is an important mission for contemporary cognitive psychology. Although the effects of quizzing and review are small, they are consistent and specific to the learning materials.

## Background

Undergraduate university students have only a fragmentary knowledge about effective learning strategies (Bjork, Dunlosky, & Kornell, 2013; Karpicke, Butler, & Roediger, 2009; Putnam, Sunkhasattee, & Roediger, 2016). In contrast, cognitive psychologists have a fine-grained understanding about strategies and activities that benefit learning in laboratory studies. For example, there is convincing evidence that testing is a very effective learning strategy (Roediger & Karpicke, 2006). In fact, even unsuccessful retrieval attempts can enhance subsequent learning (Kornell, Hays, & Bjork, 2009; Richland, Kornell, & Kao, 2009). In order to test whether these findings transfer to undergraduate university student learning in a classroom setting, the present study used a brief quiz at the beginning of each lecture on the topic to be covered in that particular lecture. Beside this intervention, which focused on retrieval before the lecture, a second intervention was administered that focused on retrieval after the lecture. Specifically, the students were given the opportunity to obtain an extra point towards the final exam by reviewing the key points of each lecture on a regular basis.

There is already some evidence that quizzing as a learning tool can be successfully used in the classroom. For example, regularly answering a set of questions that required the retrieval of information from the same day’s class significantly improved subsequent exam performance in a statistics course (Lyle & Crawford, 2011). In an introductory psychology class, Pennebaker, Gosling, and Ferrell (2013) presented a brief computerized quiz using an online system to test for the content of the previous lesson. Their results showed better performance and moreover, a reduction in the gap between students from different social classes. Using an experimental approach in the classroom, Roediger, Agarwal, McDaniel, and McDermott (2011) showed that quizzing had a beneficial effect for middle school students in a social studies course. Similarly, McDaniel, Agarwal, Huelser, McDermott, and Roediger (2011) demonstrated that quizzing improved learning in an eighth-grade science class. In one experiment, they specifically manipulated the placement of quizzing, with some students being tested before the respective lecture was introduced in class. The results showed that, overall, review quizzing produced the greatest increase in final exam performance, advance quizzing also seemed to provide a benefit compared to not having a quiz at all. In the present study, the impact of advance quizzing is addressed in university students, notably without a strict requirement for students to complete every activity.

Although it may seem counterintuitive to answer questions about topics that have not been studied yet, answering questions beforehand activates related knowledge about the topic and makes it easier to connect new information to what is already known. Moreover, it can raise the interest in the topic and thus enhance attention during the lecture (Richland et al., 2009). Thus, it can have both direct and indirect effects. In the present study, four questions were prepared for each lecture on the university online learning platform. This allowed giving individual feedback at the end of the quiz. As classroom attendance was facultative, a podcast of the lecture that contained the link to the advance quiz was made available on the learning platform. By considering the access time to the quiz, it was possible to identify students who attended the class, students who used the online course materials, and those who did neither. Therefore, the study can also contribute to the question whether attending class and/or the availability of recorded lectures is associated with performance on the final exam (cf. Credé, Roch, & Kieszczynska, 2010; Bos, Groeneveld, van Bruggen, & Brand-Gruwel, 2016; Traphagan, Kucsera, & Kishi, 2010).

The study further investigated whether reviewing the material after the lecture would benefit learning. Specifically, students were motivated to review each lecture and to submit the three key points to the learning platform before the next lecture. For the regular submission, they received a bonus point towards the final exam. The idea behind this intervention was to provide the students with retrieval practice and thus to facilitate learning (cf. Roediger & Karpicke, 2006). Moreover, reviewing after a delay can also be considered as spaced practice, which is also known to facilitate learning (Cepeda, Pashler, Vul, Wixted, & Rohrer, 2006). As sleep enhances memory consolidation, the time window for submission did not open until the day after the lecture. Moreover, in line with the reconsolidation hypothesis, which proposes that when a memory is reactivated, the trace may return to a labile state after which time it is reconsolidated or restabilized, the idea was that the requirement for reconsolidation would further strengthen learning (Feld & Diekelmann, 2015).

The interventions were embedded in an introductory psychology course (i.e., “Introduction to Memory”) that students had to attend as part of the curriculum. Due to a change in the curriculum, a larger number of students – from the first and from the second year – were enrolled in the course (i.e., two study years). For the first-year students, the curriculum change involved a new testing format in which the exam was combined with another cognitive psychology introduction course (“Introduction to Perception”) which had taken place one semester earlier. For the benefit of the present study, the performance achieved in this other part of the exam could be used to test the specificity of the intervention effects.

## Method

### Settings and participants

Participants were undergraduate psychology students from the University of Bern who took the course “Introduction to Memory” as part of their curriculum. The study was conducted in agreement with the ethical guidelines of the Human Science Faculty of the University of Bern. The original number of students enrolled in the course was 673; a total of 300 first-year students and 210 second-year students took the final exam and were included in the analyses. Due to the change in the curriculum two student cohorts (i.e., first-year and second-year students) were taught together. Related to this change, the exam of the first-year students was combined with another course (“Introduction to Perception”), which had taken place one semester earlier. The interval between the last class of that course and the final exam was 26 weeks. For first-year students, performance achieved in this exam was used to test the specificity of the advance quizzing and review interventions. Consequently, the data on first- and second-year students were analysed separately.

### Measures and materials

The “Introduction to Memory” course consisted of thirteen lectures distributed across 13 weeks with one lecture per week, and was, to a large part, based on the book by Baddeley, Eysenck, and Anderson (2015). Each lecture was recorded and made available as a podcast on the online learning platform of the university (ilias.unibe.ch). Moreover, the slides for each lecture were uploaded before each lecture, in accordance with the recommendation by Putnam et al. (2016).

#### Advance quizzing

For each lecture, four multiple choice questions were prepared. These were accessible via the online learning platform. The questions covered some of the content of the actual lecture and provided immediate feedback about the correct solution (cf. Richland et al., 2009; Butler & Roediger, 2008). These were arranged in two different access windows. The first one was open during the lecture and was used as a proxy to assess classroom attendance. In the first part of each lecture, students were provided with about 5 minutes to answer these questions in class. The second window opened after the lecture and thus the quiz was also available for those students who did not attend the lecture, but studied the particular topic using the online materials (e.g., slides, podcasts etc.). For the purpose of this study, the number of completed quizzes was used. Thus, it was possible to have a maximum score of 13 both for the lecture time window (which was used as a proxy for classroom attendance) and for the time window that opened only after the lecture (which was used as a proxy for podcast use).

#### Review

In order to motivate the students to review the content of a lecture before the next one, students were given the opportunity to submit three key points via the online learning platform. In order to benefit from overnight consolidation (cf. Feld & Diekelmann, 2015), the opportunity to submit the review opened the day after the lecture and lasted until the day before the next lecture. Thus, it was possible to have a maximum of 13 completed reviews. An extra point towards the final exam was given when at least 12 reviews were submitted via the online platform. This additional point was considered to calculate the final grade. However, for the purpose of this study, it was not added to the points in the final exam.

#### Final exam

The final exam took place 3 weeks after the last lecture. It consisted of 22 multiple choice questions, which covered the content of all the lectures (i.e., 1–2 questions per topic). For the first-year students the exam was combined with the exam on the course “Introduction to Perception”, which was prepared accordingly. For each exam a maximum of 22 points was possible. As noted above, the extra point that could be gained by submitting the reviews on a regular basis was not considered to calculate the points for the final exam for the purpose of this study.

### Analysis

In the results section, first the descriptive statistics for each variable are presented. Next, a group comparison is provided to test for differences between first-year and second-year students. The main focus is on the relationship between advance quizzing, review and performance in the final exam, and correlations are provided separately for first-year and second-year students. To test the predictive value of these interventions, separate regression analyses were conducted. For each of these analyses, diagnostic statistics were run to check whether the assumptions were met. The results of the diagnostics are summarized here. Analyses of standard residuals showed that the data contained no outliers (standard residuals < 3). To test whether the data met the assumption of collinearity, VIF scores were calculated, with values between 1.1 and 1.5, indicating that multicollinearity was not a concern. The data also met the assumption of independent errors (with Durbin-Watson values = 2 +/− 0.5). Histograms of standardized residuals and the plots of standardized residuals showed that the errors were approximately normally distributed. The scatterplot of standardized residuals showed that the data also met the assumptions of homogeneity of variance and linearity. The data also met the assumption of non-zero variances. In a set of follow-up analyses, the exclusivity of the contribution of advance quizzing was assessed using a stricter criterion. Using hierarchical regression analyses, the extra contribution of advance quizzing in the classroom was tested after controlling for the influence of quizzing after the lecture and review.

## Results

**Fig. 1:**
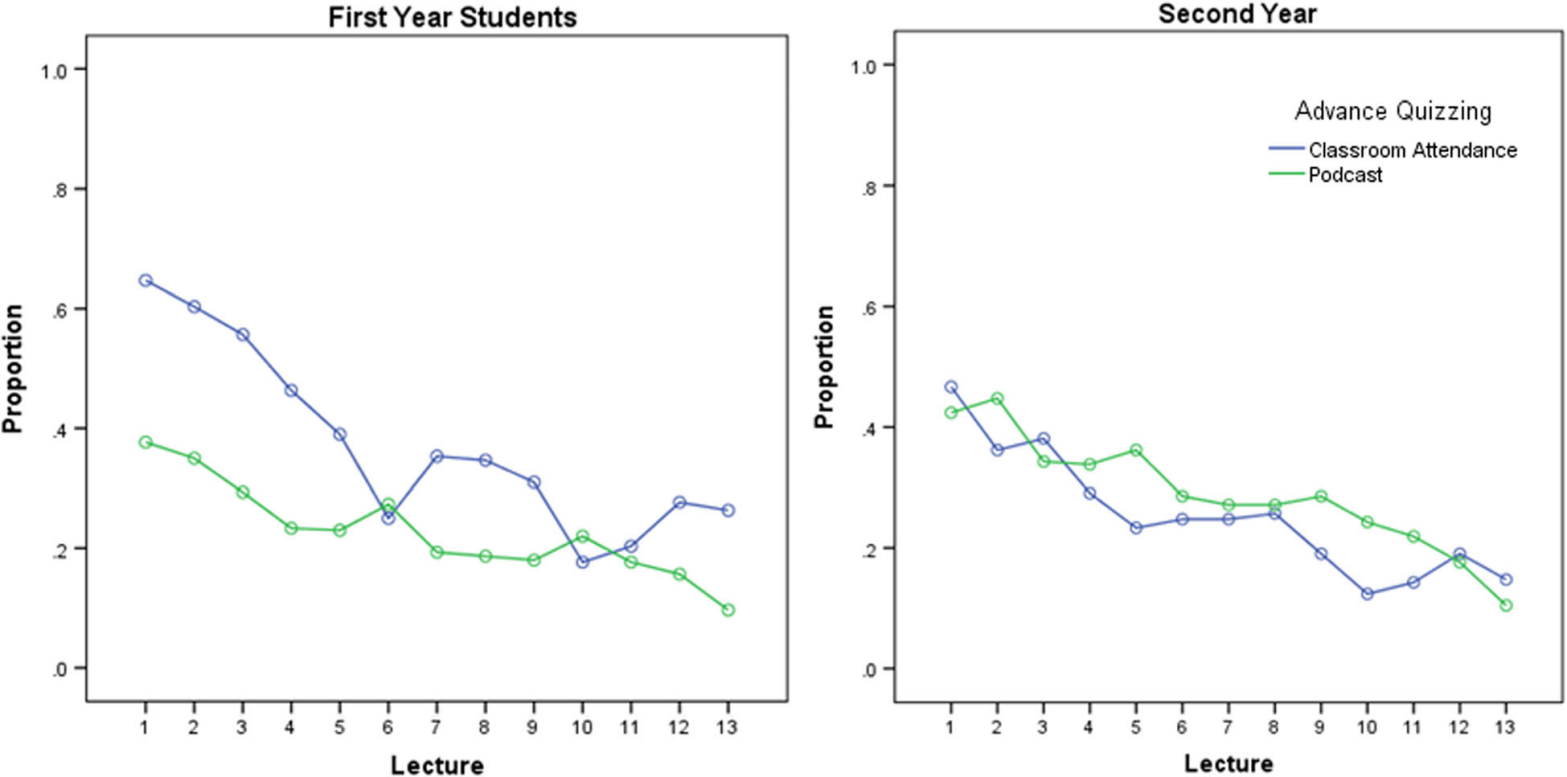
Use of advance quizzing during class (classroom attendance) or after class (podcast) across lectures and separate for first-year and second-year students

**Fig. 2:**
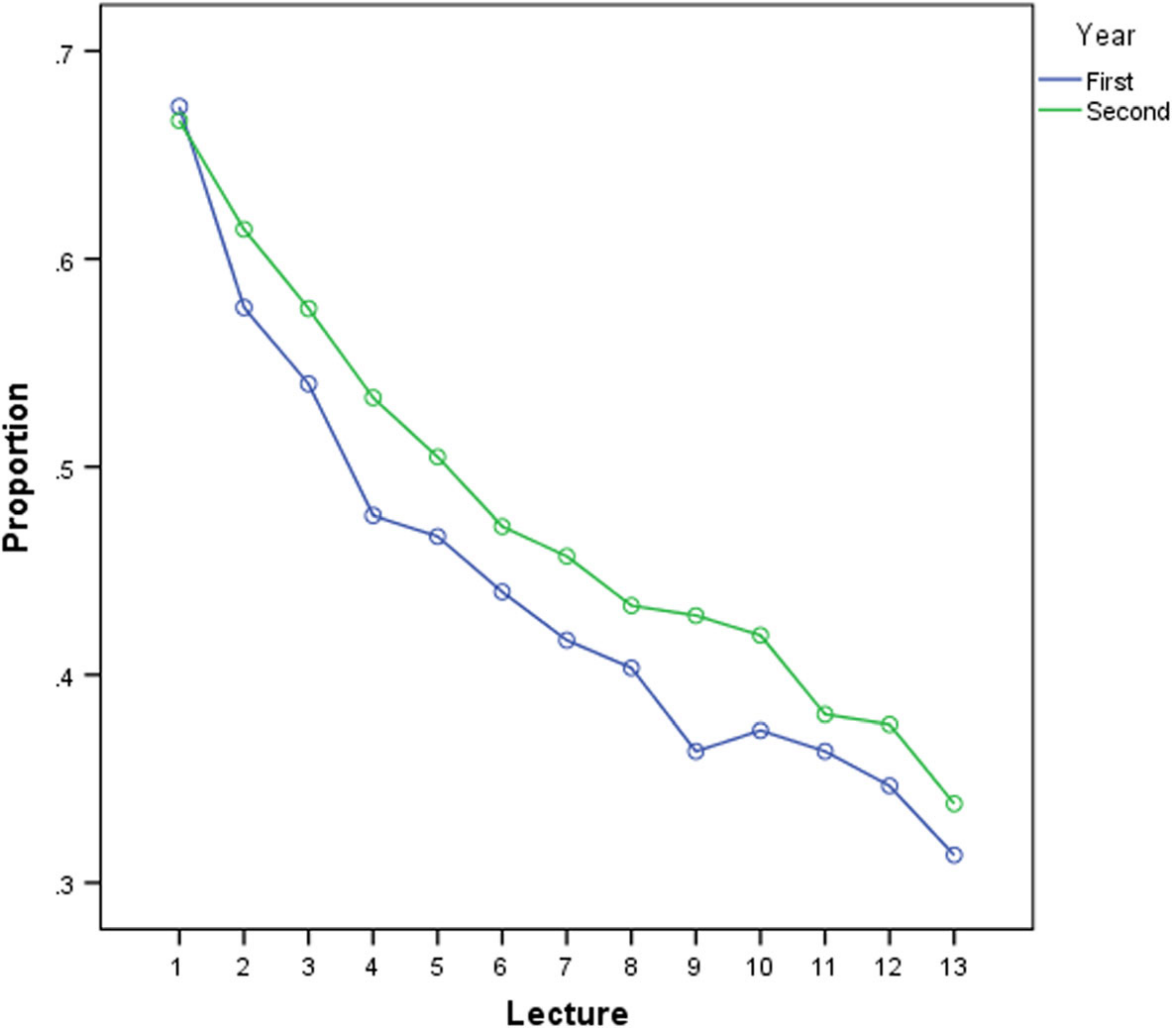
Submitted reviews of first-year and second-year students across lectures

Table 1 shows descriptive statistics for each variable. Although the main focus was on the relationship between advance quizzing, review and performance in the final exam, a first set of analyses tested for differences between the two student groups. As noted in Table 1, second-year students seemed to attend the classroom less often than first-year students. However, they used the advance quizzing opportunity that was available after the lecture more often. These results are also illustrated in Fig. 1, which depicts the number of advance quizzes that were answered in the lecture (i.e., classroom attendance) and after the lecture (i.e., podcast use). There was no group difference in the number of reviews that were submitted via the learning platform. These results are illustrated in Fig. 2. In the final exam, second-year students performed better than first-year students (Table 1). This may be due to the fact that first-year students had to complete more concurrent exams than second-year students.

**Table 1:** Descriptive statistics for advance quizzing, review and memory exam

	First year			Second year			*T*	*p*
	Mean	SD	Number	Mean	SD	Number		
Advance quizzing								
Classroom	4.8	3.9	300	3.3	3.8	210	−4.48	< .01
Podcast	3.0	3.9	300	3.8	4.4	210	2.19	< .05
Review	5.8	5.6	300	6.2	5.7	210	0.87	.38
Final exam	12.5	3.8	300	16.2	3.0	210	12.05	< .01

Advance quizzing and review reflect the number of completed assignments (out of 13). Memory exam refers to the number of points (out of 22)

In order to examine the relationship between advance quizzing, review and performance in the final exam, correlation was separately tested for first-year and second-year students, respectively. The correlation coefficients are presented in Table 2. Correlation between using advance quizzing during the class vs. using it after the class was weak in both groups. However, in both groups there were moderate correlation between advance quizzing and the scores in the final exam for the “Introduction to Memory” course, in which these interventions were introduced. Notably, correlation was weak for the “Introduction to Perception” course, in which these interventions were not implemented.

**Table 2:** Correlation between advance quizzing, review and exam scores in first-year students (normal font), and second-year students (italics), respectively

	AQ classroom	AQ podcast	Review	Memory
AQ classroom	1	*−0.178*	0.269*	0.218*
AQ podcast	0.037	1	0.366*	0.221*
Review	0.341*	0.441*	1	0.356*
Memory exam	0.230*	0.329*	0.305*	1
Perception exam	0.168	0.136	0.174	0.595*

*AQ* Advance quizzing

*p < .001

To test the predictive value of these interventions, three separate regression analyses were conducted. The first analysis focused on the first-year students. The second analysis focused on the second-year students to establish the generality of the results. The third analysis focused on the specificity of the influences of advance quizzing and review by testing whether the interventions would also predict the performance of the first-year students in the “Introduction to Perception” course.

The first analysis which focused on the first-year students included advance quizzing in the classroom, and after class (supposedly when using the podcast), and review as predictors and the “Introduction to Memory” exam as the criterion. The predictor variables explained a significant amount of variance of the criterion variable, *F*(3, 296) = 19.84, *p* < .001, *R* ^*2*^ = .167. The regression coefficients are presented in Table 3. They show that advance quizzing in the classroom, quizzing after class, and review all contributed significantly to predict the exam score.

**Table 3:** Regression analysis predicting “Introduction to Memory” exam performance from advance quizzing and review in first-year students

Variable	b	SE B	Beta	*T*	*p*
Constant	10.411	3.550		29.321	< .001
Advance quizzing					
Classroom	0.171	0.055	.177	3.110	.002
Podcast	0.258	0.058	.267	4.472	< .001
Review	0.085	0.042	.127	2.009	.045

*R* ^*2*^ = .167

The second analysis focused on the second-year students and included the same variables. Again, the predictors explained a significant amount of variance in the criterion variable, *F*(3, 206) = 13.41, *p* < .001, *R* ^*2*^ = .163. The regression coefficients are presented in Table 4. Again, advance quizzing in the classroom, quizzing after class, and review all contributed significantly to predict the exam score, thus replicating the findings from the first-year students.

**Table 4:** Regression analysis predicting “Introduction to Memory” exam performance from advance quizzing and review in second-year students

Variable	b	SE B	Beta	*T*	*p*
Constant	14.547	0.337		43.152	< .001
Advance quizzing					
Classroom	0.143	0.055	.180	2.594	.010
Podcast	0.114	0.050	.163	2.262	.025
Review	0.131	0.039	.248	3.365	.001

*R* ^*2*^ = .163

To test for the specificity of the interventions, the “Introduction to Perception” exam was used as the dependent variable in the third analysis. If the results of the interventions found in the first two regression analyses are not simply artefacts, then the amount of variance explained should be much smaller. The results showed that the predictor variables still explained a significant amount of variance in the criterion variable, *F*(3, 296) = 5.23, *p* < .01, *R* ^*2*^ = .050. However, the amount of explained variance was low (i.e., only 5% compared to 16–17% for the memory exam). As can be seen in Table 5, only the regression coefficient of advance quizzing in the classroom had a significant effect. Neither advance quizzing after class nor review contributed significantly to predict the exam score. This demonstrates the specificity of the effects for the memory course.

**Table 5:** Regression analysis predicting “Introduction to Perception” exam performance from advance quizzing and review of “introduction to memory” in first-year students

Variable	b	SE B	Beta	*T*	*p*
Constant	12.678	0.362		35.034	< .001
Advance quizzing					
Classroom	0.124	0.056	.134	2.208	.028
Podcast	0.085	0.059	.092	1.446	.149
Review	0.056	0.043	.088	1.300	.195

*R* ^*2*^ = .050

If one considers classroom attendance as a stable trait of a person (e.g., conscientiousness) and if only classroom attendance would predict exam sores, one would expect the same predictive power of advance quizzing in the classroom variable for both the memory and the perception exam. However, a comparison of the standardized beta coefficients shows that the predictive power of this variable is higher for the memory exam, that is, .177 (Table 3) and .180 (Table 4) for first-year and second-year students, respectively, compared to .134 for the perception exam (Table 5). This indicates that not only classroom attendance contributes to this relationship, but also that advance quizzing in the classroom provides additional explanatory power.

### Follow-up analysis

The previous analyses evaluated the impact of the interventions assuming that the advance quizzes were always used before learning. However, it is possible that the students who did not attend class may have completed the quizzes after they studied. If so, then the quizzes would not be advance quizzes for those students. It is also possible that students accessed the quizzes during class time and used them as study guides during the lecture rather than using them at the beginning of the lecture as instructed. Again, if so, the quizzes would not be advance quizzes for those students. In order to control for these possibilities further analyses were conducted. In these analyses, the focus was on those students who completed the advance quizzes during class, and specifically, during the first part of the lecture as instructed. The latter was possible due to the availability of the exact time stamp of the access to the advance quiz. To allow for a proper measurement of the impact of advance quizzing at the beginning of the lecture, instances in which students also accessed the advance quiz after the lecture were excluded. As no time stamp information was available for the access to the podcast it was not possible to apply the same restrictions to those students who did not attend class. With these restrictions, the mean number of uses of advance quizzing during the lecture was 3.91 (SD = 3.65) for first-year students and 2.79 (SD = 3.60) for second-year students, which was statistically significant, *t*(508) = 3.44, *p* < .01.

To test whether advance quizzing during the lecture had an exclusive predictive value for the final exam, hierarchical regression analyses were conducted that included quizzing after the lecture and review in the first step and advance quizzing at the beginning of the lecture in the second step, separately for first-year students and second-year students.

The results of these analyses are presented in Tables 6 and 7, for first-year and second-year students, respectively. Consistently, the results of these follow-up analyses showed that advance quizzing before learning contributed to performance in the final exam. Specifically, the inclusion of advance quizzing before learning led to a 16% increase in the amount of explained variance (.022/.14) for first-year students and a 26% increase of the amount of explained variance for second-year students (.036/.136), respectively. These results demonstrate that advance quizzing had an additional and independent effect on memory exam performance.

**Table 6:** Hierarchical regression analysis to assess the exclusive contribution of advance quizzing before learning to the prediction of “Introduction to Memory” exam performance in first-year students

Variable	b	SE B	Beta	*T*	*p*
Step 1					
Constant	11.039	0.296		37.231	< .001
AQ after	0.234	0.058	.242	4.034	< .001
Review	0.132	0.040	.199	3.312	.001
Step 2					
Constant	10.388	0.376		27.638	< .001
AQ after	0.319	0.065	.329	4.902	< .001
Review	0.082	0.043	.123	1.892	.059
AQ before	0.176	0.063	.171	2.767	.006

*R* ^*2*^ = .140 for Step 1; Δ *R* ^*2*^ = .022 for Step 2 (*p*s < .001)

*AQ* Advance quizzing

**Table 7:** Hierarchical regression analysis to assess the exclusive contribution of advance quizzing before learning to the prediction of “Introduction to Memory” exam performance in second-year students

Variable	b	SE B	Beta	*T*	*p*
Step 1					
Constant	14.939	0.306		48.899	< .001
AQ after	0.073	0.048	.105	1.516	.131
Review	0.168	0.037	.317	4.569	< .001
Step 2					
Constant	14.430	0.344		41.901	< .001
AQ after	0.143	0.053	.205	2.706	.007
Review	0.126	0.039	.237	3.242	.001
AQ before	0.183	0.061	.216	3.002	.003

*R* ^2^ = .136 for Step 1; Δ *R* ^2^ = .036 for Step 2 (ps < .001)

*AQ* Advance quizzing

## Discussion

The purpose of the present study was to investigate whether retrieval practice before learning (i.e., advance quizzing) and retrieval practice after learning (i.e., review between lectures) would affect final exam grades in an introductory psychology class. The results indicated that both variables enhanced final grades. Although the effects were small, they were consistent and replicated in two separate student groups (i.e., first-year and second-year students). Moreover, the effect was specific as it was not present in another cognitive psychology introduction course attended by the same first-year students.

These results are in line with other studies that showed beneficial effects of quizzing, retrieval practice, and spaced retrieval (e.g., McDaniel et al., 2011; Richland et al., 2009; Roediger & Karpicke, 2006). It is likely that these interventions have both direct and indirect effects on final exam performance. The specific processing of study materials can lead to greater familiarity and reduce anxiety in particular for complex materials. Quizzing and retrieval can lead to more elaborate associations and retrieval cues, can enhance consolidation, and lead to more elaborate memory traces through reconsolidation. Repeated processing of the materials can also lead to retrieval-induced facilitation for other materials and enhanced transfer of learning (Chan, McDermott, & Roediger, 2006; Rohrer, Taylor, & Sholar, 2010). Advance quizzing may specifically help in building up new memory representations. It may function as an advance organizer that guides attention through the lectures and thus enhances attentiveness. Importantly, due to the voluntary nature of the interventions in this study, self-initiated processes may be particularly boosted. The interventions can also help to optimize students’ learning strategies, e.g., helping students identify what they do not understand, which in turn can trigger further studying, and guide them to schedule their study time (Bjork et al., 2013; Hartwig & Dunlosky, 2012; Karpicke et al., 2009; Putnam et al., 2016).

Overall, the results of the current study indicate that even rather simple interventions based on the insights from basic research in laboratory cognitive psychology can have a significant impact on student learning in the classroom. The results confirm theoretical considerations from laboratory studies on the effects of testing, pretesting, and the forward effect of testing (e.g., Roediger & Karpicke, 2006, Kornell et al., 2009; Richland et al., 2009; Pastötter & Bäuml, 2014). In contrast to previous work, in the present study, the interventions were conceptualized on a voluntary basis, that is, students were not strictly required to participate in the interventions. Thus, the quality of the submitted responses, in particular for the review intervention, may have varied substantially. Nevertheless, participating on a regular basis still improved overall course performance.

The results also shed light on whether or not classroom attendance is necessary for successful performance. They indicate that classroom presence is not a mandatory precondition for success in the final exam. Rather it seems that using the provided materials on the online platform such as the podcast and slides can compensate for the lack of “live” experience. These results are in line with other studies that have found no detrimental effects of new technologies compared to traditional face-to-face lectures (e.g., Bos et al., 2016; Grabe & Christopherson, 2008). They are also encouraging for the growing field of distance education where classroom attendance is not possible at all.

However, a limitation of the present study is the measurement of classroom attendance. It is important to note that advance quizzing in the classroom was used as a proxy for classroom attendance. As some students did not bring their computers/smartphones to class, this measure is not comprehensive. Similarly, using advance quizzing after the class as a proxy for podcast use is also imprecise. In fact, the specific use of podcasts was not assessed at all. Nevertheless, the results indicate that advance quizzing during class and advance quizzing after class both contributed to predicting the final exam performance.

On a related note, it is possible that students who did not attend class may have completed the quizzes after they studied. If so, then the quizzes would not be advance quizzes for those students. Similarly, it is possible that students accessed the quizzes during class time and used them as study guides during the lecture rather than using them before learning. If so, the quizzes would not be advance quizzes for those students. In order to control for these possibilities further analyses were conducted to test for the independent contribution of *advance* quizzing. In these analyses, the focus was on those students who completed the advance quizzes during class, and specifically, during the first part of the lecture as instructed. In addition, instances in which students also accessed the advance quiz after the lecture were excluded. The results of these analyses showed that advance quizzing before learning contributed exclusively to performance in the final exam over and above the other two variables.

A caveat may also apply to the review intervention. As the specific review submissions were not controlled for quality or accuracy, they also represent a rather vague variable. It is very possible that the predictive value of review would have been even stronger if such a quality control had been implemented. This may be an avenue for future research. Despite these shortcomings, the presence of a consistent enhancing effect is striking.

Finally, due to the correlational approach, it is likely that extraneous variables such as individual differences also influenced the outcome. Specifically, more conscientious students may study more, may be more likely to participate in the offer of learning aids, and may attend class more often. Although the correlation between advance quizzing and final exam performance was much stronger for the “Introduction to Memory” class than for the “Introduction to Perception” class, the latter correlation was still significant. This supports the hypothesis that individual differences also contribute to the learning outcomes.

## Conclusions

To conclude, this study demonstrates that simple cognitive interventions transfer from laboratory to classroom settings. Retrieval benefits learning even for materials to be studied but not yet studied (i.e., advance quizzing). Retrieval induced as a review of key points between lectures also benefits learning. Transferring these strategies and activities into the classroom is an important mission for contemporary cognitive psychology. The results indicate that some effective interventions can be implemented rather easily into the lectures to boost student learning.

## Additional file

Additional file 1: Optimizing learning: Raw data. (XLSX 72 kb)
